# Tetra­aqua­bis­(1*H*-benzimidazole-5,6-di­carboxyl­ato-κ*N* ^3^)cobalt(II) dimethyl­formamide disolvate dihydrate

**DOI:** 10.1107/S1600536809043177

**Published:** 2009-10-23

**Authors:** Hao Wang, Wen-Dong Song, Shi-Jie Li, Dong-Liang Miao, Jin Liu

**Affiliations:** a College of Food Science and Technology, Guang Dong Ocean University, Zhanjiang 524088, People’s Republic of China; b College of Science, Guang Dong Ocean University, Zhanjiang 524088, People’s Republic of China

## Abstract

In the mononuclear title compound, [Co(C_9_H_4_N_2_O_4_)_2_(H_2_O)_4_]·2C_3_H_7_NO·2H_2_O, the Co^II^ atom, which lies on a center of inversion, is coordinated by four water mol­ecules and two N atoms from two two symmetry-related 1*H*-benzimidazole-5,6-dicarboxyl­ate ligands in a distorted octa­hedral geometry. The packing is governed by inter­molecular O—H⋯O and N—H⋯O hydrogen-bonding inter­actions.

## Related literature

For 1*H*-benzimidazole-5,6-dicarboxyl­ate complexes, see: Song *et al.* (2009*a* ▶,*b* ▶,*c* ▶).
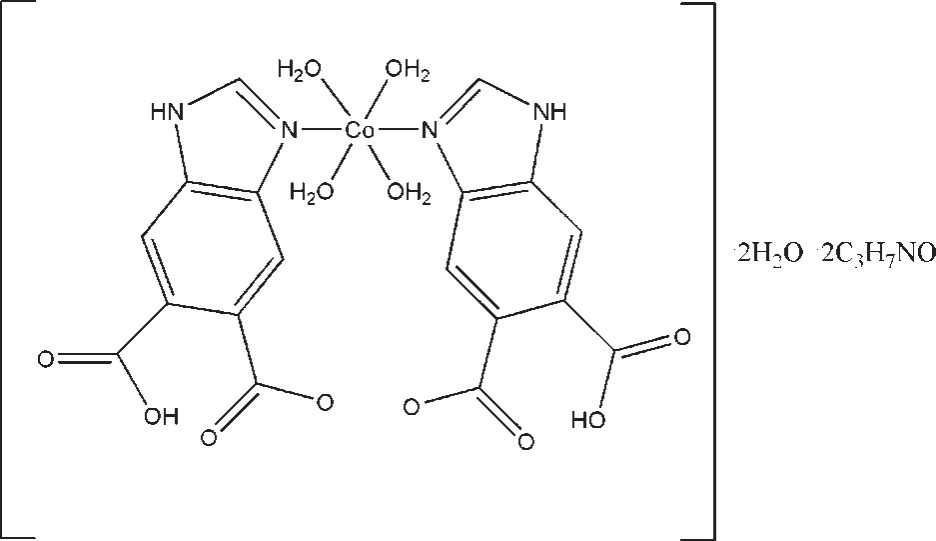


## Experimental

### 

#### Crystal data

[Co(C_9_H_5_N_2_O_4_)_2_(H_2_O)_4_]·2C_3_H_7_NO·2H_2_O
*M* *_r_* = 723.52
Triclinic, 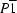

*a* = 8.5612 (17) Å
*b* = 9.1475 (18) Å
*c* = 11.642 (2) Å
α = 100.82 (3)°
β = 102.98 (3)°
γ = 114.11 (3)°
*V* = 769.9 (3) Å^3^
*Z* = 1
Mo *K*α radiation
μ = 0.64 mm^−1^
*T* = 293 K
0.30 × 0.24 × 0.21 mm

#### Data collection

Rigaku/MSC Mercury CCD diffractometer
Absorption correction: multi-scan (*REQAB*; Jacobson, 1998 ▶) *T*_min_ = 0.831, *T*_max_ = 0.877
6161 measured reflections
2751 independent reflections
2594 reflections with *I* > 2σ(*I*)
*R*_int_ = 0.020

#### Refinement

*R*[*F* ^2^ > 2σ(*F* ^2^)] = 0.038
*wR*(*F* ^2^) = 0.127
*S* = 0.96
2751 reflections
219 parameters
10 restraints
H atoms treated by a mixture of independent and constrained refinement
Δρ_max_ = 0.58 e Å^−3^
Δρ_min_ = −0.44 e Å^−3^

### 

Data collection: *RAPID-AUTO* (Rigaku, 1998 ▶); cell refinement: *RAPID-AUTO*; data reduction: *CrystalStructure* (Rigaku/MSC, 2002 ▶); program(s) used to solve structure: *SHELXS97* (Sheldrick, 2008 ▶); program(s) used to refine structure: *SHELXL97* (Sheldrick, 2008 ▶); molecular graphics: *ORTEPII* (Johnson, 1976 ▶); software used to prepare material for publication: *SHELXL97*.

## Supplementary Material

Crystal structure: contains datablocks I, global. DOI: 10.1107/S1600536809043177/ng2669sup1.cif

Structure factors: contains datablocks I. DOI: 10.1107/S1600536809043177/ng2669Isup2.hkl

Additional supplementary materials:  crystallographic information; 3D view; checkCIF report

## Figures and Tables

**Table 1:** Hydrogen-bond geometry (Å, °)

*D*—H⋯*A*	*D*—H	H⋯*A*	*D*⋯*A*	*D*—H⋯*A*
O2—H2⋯O5^i^	0.839 (10)	1.750 (11)	2.588 (3)	177 (5)
O3*W*—H6*W*⋯O1^ii^	0.84	1.98	2.800 (3)	165
O3*W*—H5*W*⋯O3^iii^	0.84	1.85	2.686 (3)	179
O2*W*—H3*W*⋯O3^iv^	0.84	1.80	2.636 (3)	174
O2*W*—H4*W*⋯O3*W*	0.84	2.06	2.811 (3)	148
O1*W*—H1*W*⋯O4^iv^	0.84	1.79	2.624 (3)	170
O1*W*—H2*W*⋯O3*W*^v^	0.84	1.94	2.749 (3)	161
N1—H1⋯O5^ii^	0.86	1.98	2.782 (3)	154
N1—H1⋯O5^ii^	0.86	1.98	2.782 (3)	154

Symmetry codes: (i) 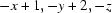; (ii) 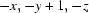; (iii) 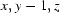; (iv) 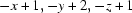; (v) 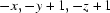.

**Fig. 1.**
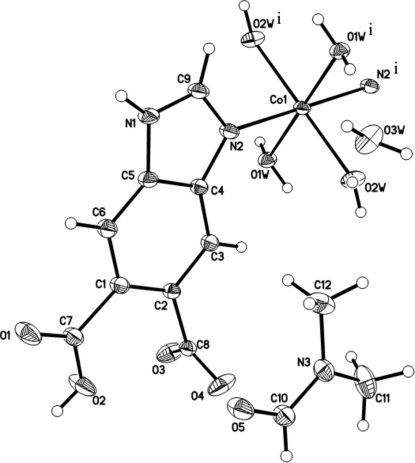
The structure of the title compound, showing the atomic numbering scheme with 30% probability displacement ellipsoids. [Symmetry codes: (i) -x, 1 - y, 1 - z.]

**Fig. 2.**
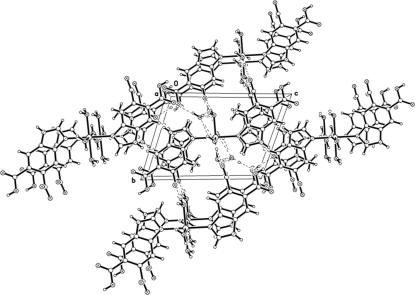
A view of the three-dimensional network constructed by O—H···O and N—H···O hydrogen bonding interactions.

## supplementary crystallographic information

### Comment

From the structuralpoint of view, 1*H*-benzimidazole-5,6-dicarboxylic acid possesses two nitrogen atoms of imidazole ring and four oxygen atoms of carboxylate groups, and might be used as versatile linker in constructing coordination polymers with abundant hydrogen bonds. And based on this idea a series of coordination polymers fomed by this ligand have been reported by us: Pentaaqua(1*H*-benzimidazole-5,6- dicarboxylato-kN^3^)cobalt(II)pentahydrate (Song *et al.*, 2009b), Pentaaqua(1*H*-benzimidazole-5,6-dicarboxylato-κN^3^)nickel(II)pentahydrate (Song *et al.*, 2009c), *catena*-Poly[[diaqua(1,10-phenanthroline-k^2^N,*N*') nickel(II)]-µ-1*H*-benzimidazole-5,6-dicarboxylato-k^2^N^3^:*O*^6^] (Song *et al.*, 2009a).In the present paper, we synthesized a novel coordination complex [Co(C_9_H_4_N_2_O_4_)_2_(H_2_O)_4_].2H_2_O.2C_3_H_7_NO.

As shown in Figure 1, the Co^II^ atom exhibits an octahedral coordination sphere, defined by two N atoms from two different 1*H*-benzimidazole-5,6-dicarboxylate ligands, and four water molecules. The equatorial plane is defined by O1w, O2w, O1w^i^ and O2w^i^ atoms, while N1 and N1^i^ occupy the axial position (symmetry codes: i = -*x*, 1 - *y*, 1 - *z*). The solvent (water and dimethylformamide) molecules are also present in the asymmetic unit. Inter/intramolecular O—H···O and N—H···O hydrogen bonds form a three-dimensional supramolecular network making the structure more stable(Fig 2).The hydrogen bonds are in the normal range(Table 1).

### Experimental

A C_3_H_7_NO solution (20 mL)containing Co(NO_3_)_2_(0.1 mmol)and 1*H*-benzimidazole-5,6-dicarboxylic acid(0.2 mmol) was stirred for a few minutes in air,and left to stand at room temperature for about a few weeks, then the red crystals were obtained.

### Refinement

Carbon and nitrogen bound H atoms were placed at calculated positions and were treated as riding on the parent C or N atoms with C—H = 0.93 Å, N—H = 0.86 Å, and with *U*_iso_(H) = 1.2 *U*_eq_(C, N). The water H-atoms were located in a difference map, and were refined with a distance restraint of O—H = 0.84 Å; their *U*_iso_ values were refined.

### Crystal data

**Table:**

[Co(C_9_H_5_N_2_O_4_)_2_(H_2_O)_4_]·2C_3_H_7_NO·2H_2_O	*Z* = 1
*M**_r_* = 723.52	*F*(000) = 377
Triclinic, *P*1	*D*_x_ = 1.561 Mg m^−^^3^
Hall symbol: -P 1	Mo *K*α radiation, λ = 0.71073 Å
*a* = 8.5612 (17) Å	Cell parameters from 3420 reflections
*b* = 9.1475 (18) Å	θ = 3.3–27.4°
*c* = 11.642 (2) Å	µ = 0.64 mm^−^^1^
α = 100.82 (3)°	*T* = 293 K
β = 102.98 (3)°	Block, red
γ = 114.11 (3)°	0.30 × 0.24 × 0.21 mm
*V* = 769.9 (3) Å^3^	

### Data collection

**Table:**

Rigaku/MSC Mercury CCD diffractometer	2751 independent reflections
Radiation source: fine-focus sealed tube	2594 reflections with *I* > 2σ(*I*)
graphite	*R*_int_ = 0.020
ω scans	θ_max_ = 25.2°, θ_min_ = 3.3°
Absorption correction: multi-scan (*REQAB*; Jacobson, 1998)	*h* = −10→10
*T*_min_ = 0.831, *T*_max_ = 0.877	*k* = −10→10
6161 measured reflections	*l* = −13→13

### Refinement

**Table:**

Refinement on *F*^2^	Primary atom site location: structure-invariant direct methods
Least-squares matrix: full	Secondary atom site location: difference Fourier map
*R*[*F*^2^ > 2σ(*F*^2^)] = 0.038	Hydrogen site location: inferred from neighbouring sites
*wR*(*F*^2^) = 0.127	H atoms treated by a mixture of independent and constrained refinement
*S* = 0.96	*w* = 1/[σ^2^(*F*_o_^2^) + (0.0795*P*)^2^ + 1.194*P*] where *P* = (*F*_o_^2^ + 2*F*_c_^2^)/3
2751 reflections	(Δ/σ)_max_ < 0.001
219 parameters	Δρ_max_ = 0.58 e Å^−^^3^
10 restraints	Δρ_min_ = −0.44 e Å^−^^3^

### Special details

**Table:**

Geometry. All e.s.d.'s (except the e.s.d. in the dihedral angle between two l.s. planes) are estimated using the full covariance matrix. The cell e.s.d.'s are taken into account individually in the estimation of e.s.d.'s in distances, angles and torsion angles; correlations between e.s.d.'s in cell parameters are only used when they are defined by crystal symmetry. An approximate (isotropic) treatment of cell e.s.d.'s is used for estimating e.s.d.'s involving l.s. planes.
Refinement. Refinement of *F*^2^ against ALL reflections. The weighted *R*-factor *wR* and goodness of fit *S* are based on *F*^2^, conventional *R*-factors *R* are based on *F*, with *F* set to zero for negative *F*^2^. The threshold expression of *F*^2^ > σ(*F*^2^) is used only for calculating *R*-factors(gt) *etc*. and is not relevant to the choice of reflections for refinement. *R*-factors based on *F*^2^ are statistically about twice as large as those based on *F*, and *R*- factors based on ALL data will be even larger.

### Fractional atomic coordinates and isotropic or equivalent isotropic displacement parameters (Å^2^)

**Table:**

	*x*	*y*	*z*	*U*_iso_*/*U*_eq_	
C1	0.1417 (4)	0.8422 (3)	0.1200 (2)	0.0279 (6)	
C2	0.2484 (3)	0.8904 (3)	0.2467 (2)	0.0247 (5)	
C3	0.1906 (3)	0.7887 (3)	0.3191 (2)	0.0269 (5)	
H3	0.2595	0.8201	0.4020	0.032*	
C4	0.0277 (3)	0.6389 (3)	0.2654 (2)	0.0248 (5)	
C5	−0.0742 (3)	0.5940 (3)	0.1405 (2)	0.0272 (6)	
C6	−0.0194 (4)	0.6932 (4)	0.0667 (2)	0.0309 (6)	
H6	−0.0888	0.6606	−0.0163	0.037*	
C7	0.1921 (4)	0.9449 (4)	0.0363 (3)	0.0324 (6)	
C8	0.4297 (3)	1.0478 (3)	0.3103 (2)	0.0263 (5)	
C9	−0.2117 (4)	0.4034 (3)	0.2225 (3)	0.0310 (6)	
H9	−0.2984	0.3064	0.2299	0.037*	
C10	0.6732 (4)	0.7964 (4)	0.1713 (3)	0.0344 (6)	
H10	0.7801	0.8972	0.1955	0.041*	
N3	0.6382 (3)	0.7242 (3)	0.2550 (2)	0.0340 (5)	
Co1	0.0000	0.5000	0.5000	0.02238 (18)	
N1	−0.2262 (3)	0.4426 (3)	0.1168 (2)	0.0315 (5)	
H1	−0.3137	0.3846	0.0473	0.038*	
N2	−0.0630 (3)	0.5150 (3)	0.3148 (2)	0.0282 (5)	
C12	0.4674 (5)	0.5710 (5)	0.2251 (4)	0.0552 (9)	
H3A	0.3742	0.6008	0.2330	0.083*	
H3B	0.4829	0.5094	0.2812	0.083*	
H3C	0.4330	0.5021	0.1414	0.083*	
C11	0.7568 (6)	0.8059 (5)	0.3848 (3)	0.0547 (9)	
H4A	0.8640	0.9036	0.3911	0.082*	
H4B	0.7907	0.7283	0.4145	0.082*	
H4C	0.6937	0.8393	0.4340	0.082*	
O1	0.1079 (3)	0.8972 (3)	−0.0726 (2)	0.0582 (7)	
O2	0.3337 (4)	1.0933 (3)	0.0904 (2)	0.0635 (8)	
O3	0.4308 (3)	1.1767 (3)	0.3708 (2)	0.0458 (6)	
O4	0.5660 (3)	1.0359 (3)	0.3035 (3)	0.0524 (6)	
O5	0.5725 (3)	0.7391 (3)	0.06005 (19)	0.0457 (6)	
O1W	0.0884 (2)	0.7574 (2)	0.57397 (17)	0.0300 (4)	
H2W	0.0296	0.7784	0.6176	0.045*	
H1W	0.2007	0.8133	0.6126	0.045*	
O2W	0.2619 (3)	0.5455 (2)	0.50191 (19)	0.0360 (5)	
H4W	0.2576	0.4881	0.4347	0.054*	
H3W	0.3630	0.6321	0.5381	0.054*	
O3W	0.1590 (3)	0.2539 (3)	0.3083 (2)	0.0498 (6)	
H5W	0.2452	0.2312	0.3275	0.075*	
H6W	0.0905	0.2004	0.2343	0.075*	
H2	0.367 (6)	1.146 (5)	0.041 (3)	0.075*	

### Atomic displacement parameters (Å^2^)

**Table:**

	*U*^11^	*U*^22^	*U*^33^	*U*^12^	*U*^13^	*U*^23^
C1	0.0271 (13)	0.0262 (13)	0.0258 (13)	0.0076 (11)	0.0094 (10)	0.0090 (10)
C2	0.0231 (12)	0.0223 (12)	0.0259 (13)	0.0076 (10)	0.0088 (10)	0.0076 (10)
C3	0.0258 (13)	0.0260 (13)	0.0217 (12)	0.0073 (11)	0.0052 (10)	0.0071 (10)
C4	0.0247 (12)	0.0238 (12)	0.0245 (12)	0.0089 (10)	0.0098 (10)	0.0089 (10)
C5	0.0248 (13)	0.0230 (13)	0.0260 (13)	0.0052 (11)	0.0080 (10)	0.0062 (10)
C6	0.0303 (14)	0.0302 (14)	0.0224 (13)	0.0067 (12)	0.0060 (11)	0.0082 (11)
C7	0.0322 (14)	0.0304 (14)	0.0262 (14)	0.0071 (12)	0.0081 (11)	0.0111 (11)
C8	0.0232 (13)	0.0248 (13)	0.0257 (13)	0.0066 (11)	0.0064 (10)	0.0101 (11)
C9	0.0277 (14)	0.0244 (13)	0.0306 (14)	0.0026 (11)	0.0088 (11)	0.0103 (11)
C10	0.0334 (15)	0.0294 (14)	0.0290 (14)	0.0074 (12)	0.0047 (12)	0.0088 (11)
N3	0.0404 (13)	0.0350 (13)	0.0270 (12)	0.0174 (11)	0.0105 (10)	0.0122 (10)
Co1	0.0203 (3)	0.0186 (3)	0.0219 (3)	0.0040 (2)	0.00591 (19)	0.00645 (19)
N1	0.0243 (11)	0.0260 (12)	0.0229 (11)	−0.0027 (9)	0.0002 (9)	0.0055 (9)
N2	0.0271 (11)	0.0238 (11)	0.0262 (11)	0.0046 (9)	0.0083 (9)	0.0097 (9)
C12	0.052 (2)	0.057 (2)	0.060 (2)	0.0169 (18)	0.0275 (18)	0.0332 (18)
C11	0.085 (3)	0.055 (2)	0.0270 (16)	0.041 (2)	0.0084 (16)	0.0138 (15)
O1	0.0560 (15)	0.0498 (14)	0.0286 (11)	−0.0072 (11)	−0.0002 (10)	0.0191 (10)
O2	0.0580 (15)	0.0463 (14)	0.0325 (12)	−0.0183 (12)	−0.0036 (11)	0.0210 (11)
O3	0.0293 (11)	0.0302 (11)	0.0569 (14)	0.0051 (9)	0.0109 (10)	−0.0062 (10)
O4	0.0218 (10)	0.0340 (12)	0.0823 (18)	0.0077 (9)	0.0093 (11)	−0.0002 (11)
O5	0.0399 (12)	0.0433 (12)	0.0270 (11)	−0.0014 (10)	0.0014 (9)	0.0144 (9)
O1W	0.0238 (9)	0.0245 (9)	0.0318 (10)	0.0058 (8)	0.0057 (8)	0.0051 (8)
O2W	0.0250 (10)	0.0296 (10)	0.0399 (11)	0.0049 (8)	0.0114 (8)	0.0004 (8)
O3W	0.0428 (13)	0.0590 (15)	0.0395 (12)	0.0271 (12)	0.0088 (10)	−0.0033 (11)

### Geometric parameters (Å, °)

**Table:**

C1—C6	1.381 (4)	N3—C12	1.462 (4)
C1—C2	1.424 (4)	N3—C11	1.463 (4)
C1—C7	1.489 (4)	Co1—O1W	2.086 (2)
C2—C3	1.387 (4)	Co1—O1W^i^	2.086 (2)
C2—C8	1.513 (4)	Co1—O2W^i^	2.1007 (19)
C3—C4	1.393 (4)	Co1—O2W	2.1007 (19)
C3—H3	0.9300	Co1—N2	2.146 (2)
C4—N2	1.395 (3)	Co1—N2^i^	2.146 (2)
C4—C5	1.398 (4)	N1—H1	0.8600
C5—C6	1.382 (4)	C12—H3A	0.9600
C5—N1	1.382 (3)	C12—H3B	0.9600
C6—H6	0.9300	C12—H3C	0.9600
C7—O1	1.201 (4)	C11—H4A	0.9600
C7—O2	1.304 (4)	C11—H4B	0.9600
C8—O4	1.234 (3)	C11—H4C	0.9600
C8—O3	1.250 (3)	O2—H2	0.839 (10)
C9—N2	1.315 (4)	O1W—H2W	0.8400
C9—N1	1.338 (4)	O1W—H1W	0.8400
C9—H9	0.9300	O2W—H4W	0.8399
C10—O5	1.253 (3)	O2W—H3W	0.8399
C10—N3	1.299 (4)	O3W—H5W	0.8400
C10—H10	0.9300	O3W—H6W	0.8399
			
C6—C1—C2	120.8 (2)	O1W^i^—Co1—O2W	88.64 (8)
C6—C1—C7	115.4 (2)	O2W^i^—Co1—O2W	180.0
C2—C1—C7	123.8 (2)	O1W—Co1—N2	90.82 (9)
C3—C2—C1	120.3 (2)	O1W^i^—Co1—N2	89.18 (9)
C3—C2—C8	115.8 (2)	O2W^i^—Co1—N2	90.29 (9)
C1—C2—C8	123.9 (2)	O2W—Co1—N2	89.71 (9)
C2—C3—C4	118.9 (2)	O1W—Co1—N2^i^	89.18 (9)
C2—C3—H3	120.6	O1W^i^—Co1—N2^i^	90.82 (9)
C4—C3—H3	120.6	O2W^i^—Co1—N2^i^	89.71 (9)
C3—C4—N2	131.3 (2)	O2W—Co1—N2^i^	90.29 (9)
C3—C4—C5	119.7 (2)	N2—Co1—N2^i^	179.999 (1)
N2—C4—C5	108.9 (2)	C9—N1—C5	107.1 (2)
C6—C5—N1	132.1 (2)	C9—N1—H1	126.5
C6—C5—C4	122.4 (2)	C5—N1—H1	126.5
N1—C5—C4	105.5 (2)	C9—N2—C4	104.9 (2)
C1—C6—C5	117.9 (2)	C9—N2—Co1	124.00 (18)
C1—C6—H6	121.0	C4—N2—Co1	130.98 (18)
C5—C6—H6	121.0	N3—C12—H3A	109.5
O1—C7—O2	121.9 (3)	N3—C12—H3B	109.5
O1—C7—C1	123.1 (3)	H3A—C12—H3B	109.5
O2—C7—C1	115.0 (2)	N3—C12—H3C	109.5
O4—C8—O3	125.2 (3)	H3A—C12—H3C	109.5
O4—C8—C2	117.0 (2)	H3B—C12—H3C	109.5
O3—C8—C2	117.7 (2)	N3—C11—H4A	109.5
N2—C9—N1	113.6 (2)	N3—C11—H4B	109.5
N2—C9—H9	123.2	H4A—C11—H4B	109.5
N1—C9—H9	123.2	N3—C11—H4C	109.5
O5—C10—N3	124.3 (3)	H4A—C11—H4C	109.5
O5—C10—H10	117.8	H4B—C11—H4C	109.5
N3—C10—H10	117.8	C7—O2—H2	114 (3)
C10—N3—C12	120.6 (3)	Co1—O1W—H2W	113.1
C10—N3—C11	120.7 (3)	Co1—O1W—H1W	113.3
C12—N3—C11	118.2 (3)	H2W—O1W—H1W	110.9
O1W—Co1—O1W^i^	180.0	Co1—O2W—H4W	110.8
O1W—Co1—O2W^i^	88.64 (8)	Co1—O2W—H3W	130.9
O1W^i^—Co1—O2W^i^	91.36 (8)	H4W—O2W—H3W	112.2
O1W—Co1—O2W	91.36 (8)	H5W—O3W—H6W	112.2

Symmetry codes: (i) −*x*, −*y*+1, −*z*+1.

### Hydrogen-bond geometry (Å, °)

**Table:**

*D*—H···*A*	*D*—H	H···*A*	*D*···*A*	*D*—H···*A*
O2—H2···O5^ii^	0.84 (1)	1.75 (1)	2.588 (3)	177 (5)
O3W—H6W···O1^iii^	0.84	1.98	2.800 (3)	165
O3W—H5W···O3^iv^	0.84	1.85	2.686 (3)	179
O2W—H3W···O3^v^	0.84	1.80	2.636 (3)	174
O2W—H4W···O3W	0.84	2.06	2.811 (3)	148
O1W—H1W···O4^v^	0.84	1.79	2.624 (3)	170
O1W—H2W···O3W^i^	0.84	1.94	2.749 (3)	161
N1—H1···O5^iii^	0.86	1.98	2.782 (3)	154
N1—H1···O5^iii^	0.86	1.98	2.782 (3)	154

Symmetry codes: (ii) −*x*+1, −*y*+2, −*z*; (iii) −*x*, −*y*+1, −*z*; (iv) *x*, *y*−1, *z*; (v) −*x*+1, −*y*+2, −*z*+1; (i) −*x*, −*y*+1, −*z*+1.
